# Enterotypes in asthenospermia patients with obesity

**DOI:** 10.1038/s41598-022-20574-0

**Published:** 2022-10-10

**Authors:** Jiao Jiao, Peng Xu, Xiaobin Wang, Ze Xing, Sitong Dong, Gaoyu Li, Xinrui Yao, Renhao Guo, Tao Feng, Weifan Yao, Bochen Pan, Xuan Zhu, Xiuxia Wang

**Affiliations:** 1 Center of Reproductive Medicine, Shengjing Hospital of China Medical University, Shenyang, China; 2 Shenyang Reproductive Health Clinical Medicine Research Center, Shenyang, China; 3 SUSTC-SJH Joint Center for Human Reproduction and Genetics, Shenyang, China; 4 Germountx Company, Beijing, China; 5 School of Pharmacy, China Medical University, Shenyang, China; 6 Liaoning Key Laboratory of Molecular Targeted Anti-Tumor Drug Development and Evaluation, Shenyang, China; 7 School of Food Science and Biotechnology, Zhejiang Gongshang University, Hangzhou, China; 8 Hainan Jinghua Hejing Hospital for Reproductive Medicine, Haikou, Hainan China

**Keywords:** Microbiology, Reproductive disorders, Infertility

## Abstract

The essence of enterotypes is stratifying the entire human gut microbiome, which modulates the association between diet and disease risk. A study was designed at the Center of Reproductive Medicine, Shengjing Hospital of China Medical University and Jinghua Hospital of Shenyang. *Prevotella* and *Bacteroides* were analyzed in 407 samples of stool, including 178 men with enterotype B (61 normal, 117 overweight/obese) and 229 men with enterotype P (74 normal, 155 overweight/obese). The ratio between *Prevotella* and *Bacteroides* abundance, P/B, was used as a simplified way to distinguish the predominant enterotype. In enterotype P group (P/B ≥ 0.01), obesity was a risk factor for a reduced rate of forward progressive sperm motility (odds ratio [OR] 3.350; 95% confidence interval [CI] 1.881–5.966; P < 0.001), and a reduced rate of total sperm motility (OR 4.298; 95% CI 2.365–7.809; P < 0.001). Obesity was also an independent risk factor (OR 3.131; 95% CI 1.749–5.607; P < 0.001) after adjusting follicle-stimulating hormone. In enterotype P, body mass index, as a diagnostic indicator of a reduced rate of forward progressive sperm motility and a decreased rate of decreased total sperm motility, had AUC values of 0.627 (P = 0.001) and 0.675 (P < 0.0001), respectively, which were significantly higher than the predicted values in all patients. However, in enterotype B group (P < 0.01), obesity was not a risk factor for asthenospermia, where no significant difference between obesity and sperm quality parameters was observed. This study is tried to introduce enterotypes as a population-based individualized classification index to investigate the correlation between BMI and asthenospermia. In our study, overweight/obese men with enterotype P were found to have poorer sperm quality. however, sperm quality was not associated with overweight/obese in men with enterotype B. Thereof, BMI is a risk factor for asthenospermia only in men with enterotype P, but not in men with enterotype B.

## Introduction

Nowadays, it is generally accepted that asthenospermia, which leads to male infertility, is a complex disease in which various etiologic factors are involved. In addition to genetic factors, lifestyle, metabolic disorder, infection and environment also affect sperm motility. In recent decades, the global obesity epidemic has paralleled a decrease in sperm quality^1–7^. Therefore, obesity is considered to be a lifestyle factor that may adversely affect sperm quality. Recent studies have investigated the associations between obesity and sperm motility, but the results remain inconsistent^1,5,6,8–10^. For example, Ma et al. reported that obesity was substantially associated with a 3.6% (0.2%, 6.9%) reduction in total motile sperm count^5^. Nathalie et al. also suggested that overweight and obese men were at significantly increased odds of presenting with azoospermia compared to men of normal body weight^9^. In contrast, a prospective cohort study demonstrated that sperm quality was not statistically significantly affected by body mass index (BMI) in a cohort of male partners in sub fertile couples^8^. Taken together, the current evidence on the association between BMI and sperm quality is inconclusive. It can be speculated that that the population-based individualized differences may be involved in the effect of BMI on sperm motility.

Gut microbiota is recognized as the second genome of the human body that plays a variety of roles in health and disease affected by dietary structure. Studies have shown that a high-fat diet may lead to decreased sperm quality through alterations in intestinal microbiome^11,12^, and that modulating gut microbiota may improve sperm quality^13^.

Enterotype is a classification of gut microbiota in different populations, indicating that the variation of gut microbiota is stratified between individuals, rather than continuous. Some researches demonstrated that people with different enterotypes may have different responses to disease triggers^14^. However, the relationship between enterotype, asthenozoospermia, and BMI was not clearly demonstrated till now. In our previous researches, we found a positive relationship between asthenozoospermia and BMI, but the association was not stable in all cases. In this study, experiments were designed to investigate the association of asthenozoospermia and BMI in men with different enterotypes.

## Results

### Determination of enterotypes according to the P/B ratio

**Figure 1:**
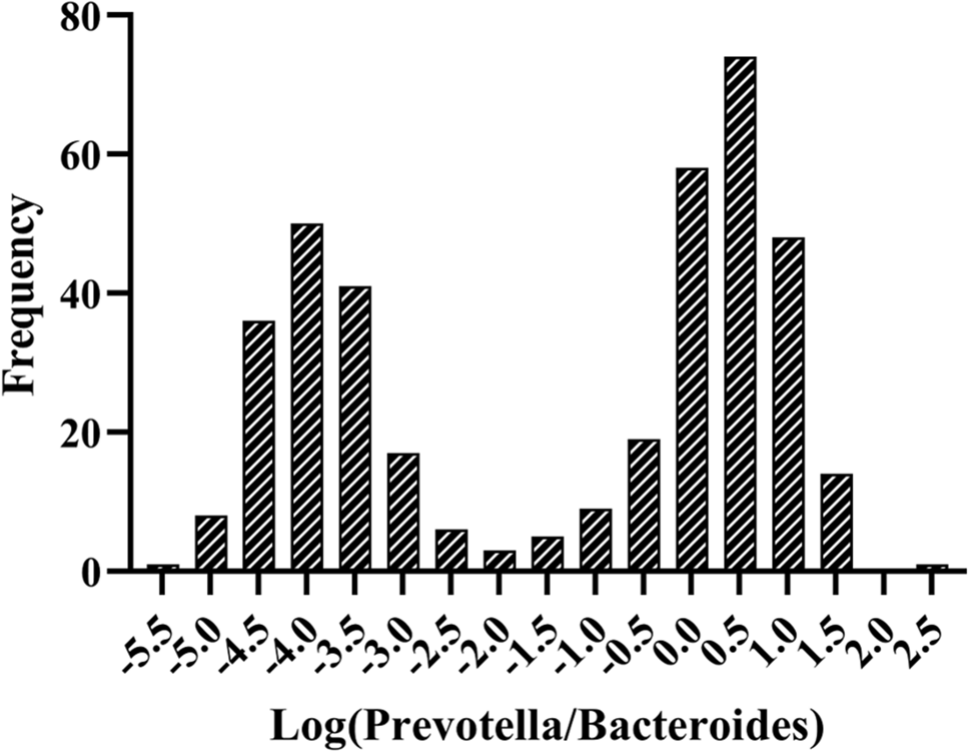
*Prevotella*/*Bacteroides* (P/B) groups. Histogram plotting frequency of the log-transformed abundance of P/B for all patients.

Gut microbiota analysis was performed on the stool samples of all participants (n = 407). The frequency plot of the relative abundance of log (P/B) (Fig. 1) demonstrated a bimodal distribution of separation between the two groups. These results suggested that the participants could be classified into two groups: enterotype P (P/B ≥ 0.01) and enterotype B (P/B < 0.01).

### Clinical parameters

The baseline characteristics, sperm quality parameters, and sex hormone data of 407 patients enrolled in this study are listed in Table 1. Patients were divided into normal (BMI < 24 kg/m^2^) group and overweight/obese (BMI ≥ 24 kg/m^2^) group according to their BMI. The baseline characteristics, sperm quality parameters, and sex hormone data of the two groups are shown in Table 2. The levels of rate of forward progressive sperm motility, rate of total sperm motility, and TT were different between the two groups. In the normal BMI group, there were more patients with normal rate of forward progressive sperm motility and total sperm motility, whereas in the overweight/obese group, there were more patients with low rate of forward progressive sperm motility and total sperm motility, and the difference was statistically significant. However, there were no statistically significant differences in total sperm count and sperm concentration between the two groups.

**Table 1:** Characteristics of participants' clinical parameters included in this study.

		***N* = 407**
**Basic information, *N* (%)**		
Age	*Median (P25, P75)*	34.00(31.00–38.00)
	< 40	349(85.7)
	≥ 40	58(14.3)
BMI	*Median (P25, P75)*	25.09(23.15–27.38)
	< 24	135(33.2)
	≥ 24	272(66.8)
**Sperm quality, *N* (%)**		
Rate of forward progressive motility	*Median (P25, P75)*	28.84(20.54–41.46)
	Astheno (< 32% motile)	234(57.5)
	Normal	173(42.5)
Rate of total motility	*Median (P25, P75)*	38.36(27.59–53.33)
	Astheno (< 40% motile)	220(54.1)
	Normal	187(45.9)
Total sperm count	*Median (P25, P75)*	174.23(95.32–280.00)
	Low (< 39 million)	11(2.7)
	Normal	396(97.3)
Sperm concentration	*Median (P25, P75)*	50.49(32.14–79.29)
	Oligo (< 15 million/mL)	11(2.7)
	Normal	396(97.3)
**Intestinal microbiome, *N* (%)**		
P/B	*Median (P25, P75)*	0.39(0.00–3.68)
	< 0.01	178(43.7)
	≥ 0.01	229(56.3)
**Sex hormone, *M*(*P25, P75*)**		
FSH	*Median (P25, P75)*	4.52(3.39–6.04)
LH	*Median (P25, P75)*	3.12(2.24–4.25)
E_2_	*Median (P25, P75)*	33.79(25.51–41.40)
PRL	*Median (P25, P75)*	6.39(4.83–8.22)
TT	*Median (P25, P75)*	3.58(2.76–4.45)

BMI, body mass index; FSH, follicle-stimulating hormone; LH, luteinizing hormone; E_2_, estradiol; TT, total testosterone; PRL, prolactin; P/B, *Prevotella*/*Bacteroides*. Median (interquartile range) are shown. The Mann–Whitney U test of nonparametric coefficients was used for non-normally distributed data.

**Table 2:** Characteristics of the normal men and the overweight/obese men.

		**BMI**		***P***
		**Normal (< 24 kg/m**^**2**^**)**	**overweight/obese(**≥ **24 kg/m**^**2**^**)**	
		***N* = 135**	***N* = 272**	
**Basic information, *N* (%)**				
Age	< 40	120(88.9)	229(84.2)	0.202
	≥ 40	15(11.1)	43(15.8)	
**Sperm quality, *N* (%)**				
Rate of forward progressive motility	Astheno (< 32% motile)	64(47.4)	170(62.5)	**0.004**
	Normal	71(52.6)	102(37.5)	
Rate of total motility	Astheno (< 40% motile)	57(42.2)	163(60.0)	**0.001**
	Normal	78(57.8)	109(40.0)	
Total sperm count	Low (< 39 million)	4(3.0)	7(2.6)	0.758
	Normal	131(97.0)	265(97.4)	
Sperm concentration	Oligo (< 15 million/mL)	4(3.0)	7(2.6)	0.758
	Normal	131(97.0)	265(97.4)	
**Intestinal microbiome,* N* (%)**				
P/B	< 0.01	61(45.2)	117(43.0)	0.678
	≥ 0.01	74(54.8)	155(57.0)	
**Sex hormone, *M*(*P25, P75*)**				
FSH		4.47(3.25–5.86)	4.56(3.44–6.09)	0.393
LH		3.17(2.35–4.36)	3.12(2.21–4.17)	0.462
E_2_		34.24(24.39–42.48)	33.75(26.21–40.07)	0.777
PRL		6.44(4.65–8.07)	6.32(4.87–8.31)	0.976
TT		4.16(3.33–5.41)	3.27(2.60–4.16)	** < 0.001**

BMI, body mass index; FSH, follicle-stimulating hormone; LH, luteinizing hormone; E_2_, estradiol; TT, total testosterone; PRL, prolactin; P/B, *Prevotella*/*Bacteroides*. Median (interquartile range) are shown. The Mann–Whitney U test of nonparametric coefficients was used for non-normally distributed data. Significant values are bold.

### Logistic regression analysis of risk factors for asthenospermia

As shown in Table 3, the incidence of asthenospermia in the overweight/obese group was significantly higher than that in the normal group (P = 0.004). Univariate logistic regression showed that decreased rate of forward progressive sperm motility (OR 1.849; 95% confidence interval [CI] 1.218–2.807; P = 0.004) and decreased rate of total sperm motility (OR 2.406; 95% CI 1.346–3.111; P = 0.001) were significantly associated with obesity, as evident from the data of the overweight/obese group. Second, multivariate logistic regression analysis confirmed that overweight/obese was an independent risk factor for decreased rate of forward progressive sperm motility (OR 1.793; 95% CI 1.177–2.729; P = 0.006) and decreased rate of total sperm motility (OR 1.981; 95% CI 1.299–3.022; P = 0.002) after adjusting for FSH (Table 3) and overweight/obese remains an independent risk factor after adjusting FSH, LH, TT and age (Supplementary Table 2).

**Table 3:** Logistic regression analysis of risk factors for asthenospermia in all participants.

	**Rate of forward progressive Motility**		***P***	**Rate of total motility**		***P***	**Total sperm count**		***P***	**Sperm concentration**		***P***
	< 32% motile	Normal		< 40% motile	Normal		< 39 million	Normal		< 15 million/mL	Normal	
	N = 234	N = 173		N = 220	N = 187		N = 11	N = 396		N = 11	N = 396	
**Age**												
< 40	196	153	**0.033**	185	164	0.299	9	340	0.661	9	340	0.661
≥ 40	38	20		35	23		2	56		2	56	
**BMI, *N***												
< 24	64	71	**0.004**	57	78	**0.001**	4	131	0.758	4	131	0.758
≥ 24	170	102		163	109		7	265		7	265	
**P/B, N**												
< 0.01	102	76	0.945	98	80	0.721	7	171	0.177	4	174	0.762
≥ 0.01	132	97		122	107		4	225		7	222	
**Sex hormone**												
FSH	4.70(3.41–6.39)	4.42(3.26–5.61)	**0.046**	4.71(3.48–6.32)	4.40(3.21–5.63)	**0.031**	7.56(5.52–9.82)	4.47(3.33–5.92)	** < 0.01**	7.18(5.52–9.82)	4.48(3.36–5.92)	**0.004**
LH	3.26(2.23–4.22)	3.03(2.24–4.27)	0.703	3.12(2.22–4.12)	3.14(2.31–4.36)	0.675	4.18(2.94–5.13)	3.11(2.23–4.19)	0.045	4.18(3.31–6.49)	3.08(2.23–4.19)	**0.004**
E_2_	33.00(24.67–41.21)	35.05(26.85–41.76)	0.149	33.58(25.29–41.86)	34.86(25.82–40.81)	0.662	26.88(23.13–47.64)	34.04(25.67–41.36)	0.371	28.13(23.13–33.79)	34.14(25.59–41.53)	0.157
PRL	6.40(4.90–8.21)	6.29(4.69–8.24)	0.845	6.49(4.94–8.27)	6.24(4.60–8.18)	0.266	6.39(4.97–7.31)	6.39(4.81–8.25)	0.758	6.39(4.86–7.31)	6.39(4.77–8.25)	0.898
TT	3.54(2.68–4.41)	3.63(2.87–4.71)	0.385	3.48(2.66–4.37)	3.67(2.88–4.65)	0.13	4.18(3.26–5.62)	3.58(2.72–4.42)	0.131	4.73(2.65–5.83)	3.57(2.76–4.41)	0.128

Curde OR	**Rate of forward progressive motility**	***P***	**Rate of total motility**	***P***	**Total sperm count**	***P***	**Sperm concentration**	***P***				
Age	1.482(0.829–2.652)	0.184										
BMI	1.849(1.218–2.807)	**0.004**	2.046(1.346–3.111)	**0.001**								
P/B												
**Sex hormone**												
FSH	0.898(0.825–0.979)	**0.014**	0.885(0.812–0.964)	**0.005**	0.825(0.726–0.938)	**0.003**	0.785(0.681–0.904)	**0.001**				
LH							0.759(0.609–0.946)	**0.014**				
E_2_												
PRL												
TT												

Adjusted OR	**Rate of forward progressive motility**	***P***	**Rate of total motility**	***P***	**Total sperm count**	***P***	**Sperm concentration**	***P***				
BMI	1.793(1.177–2.729)	**0.006**	1.981(1.299–3.022)	**0.002**								
P/B												
**Sex hormone**												
FSH	0.903(0.828–0.985)	**0.022**	0.890(0.815–0.971)	**0.009**			0.801(0.674–0.952)	**0.012**				
LH							0.944(0.705–1.265)	0.700				
E_2_												
PRL												
TT												

BMI, body mass index; FSH, follicle-stimulating hormone; LH, luteinizing hormone; E_2_, estradiol; TT, total testosterone; PRL, prolactin; P/B, *Prevotella*/*Bacteroides*. Median (interquartile range) are shown. The Mann–Whitney U test of nonparametric coefficients was used for non-normally distributed data. Significant values are bold.

### Obesity is a risk factor for asthenospermia under enterotype P

As stated, obesity is significantly associated with a decreased sperm quality. Taken account of effects of enterotypes on the risk of disease, effects of BMI on sperm quality in patients with enterotypes P and B were evaluated.

**Figure 2:**
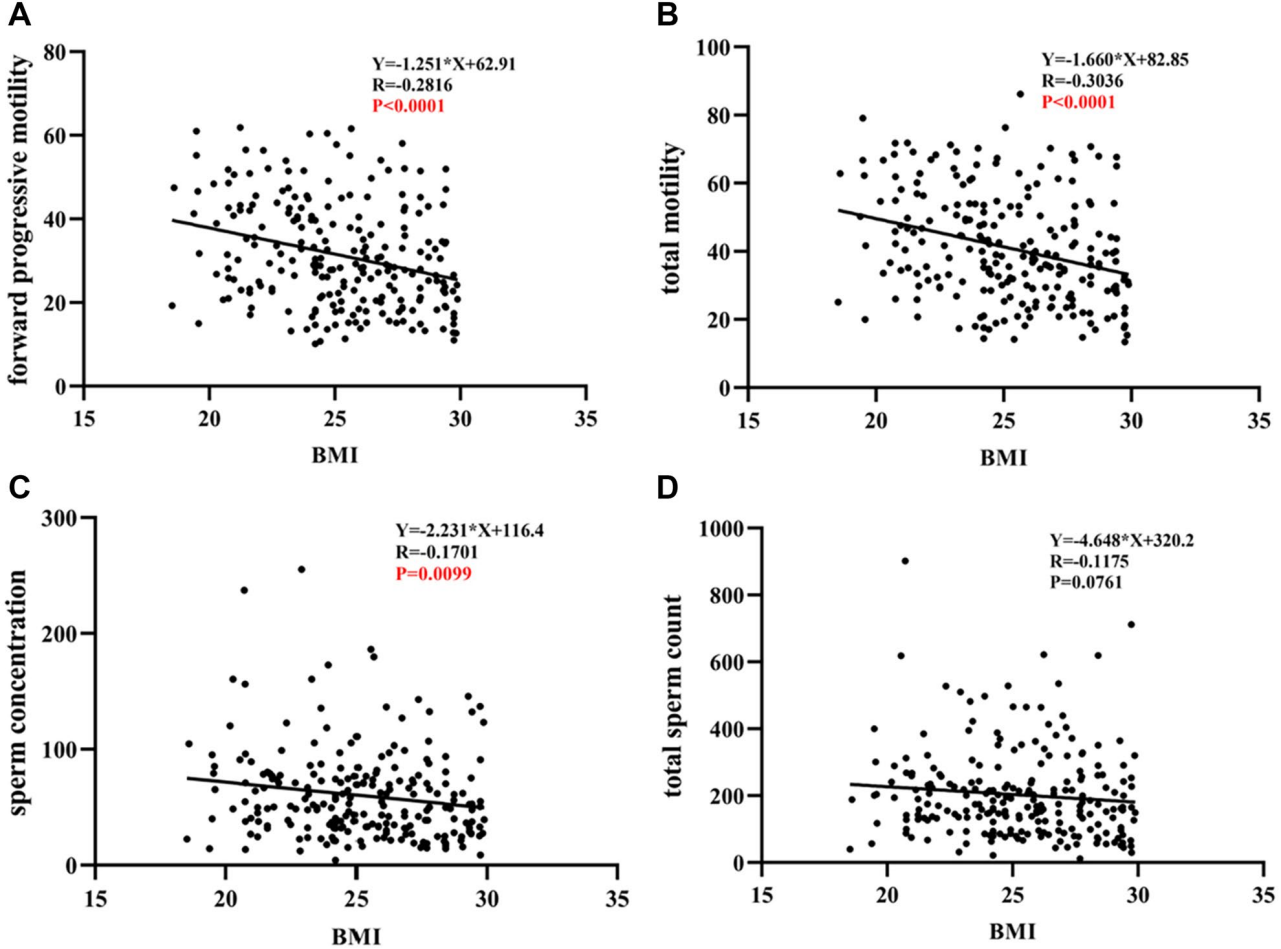

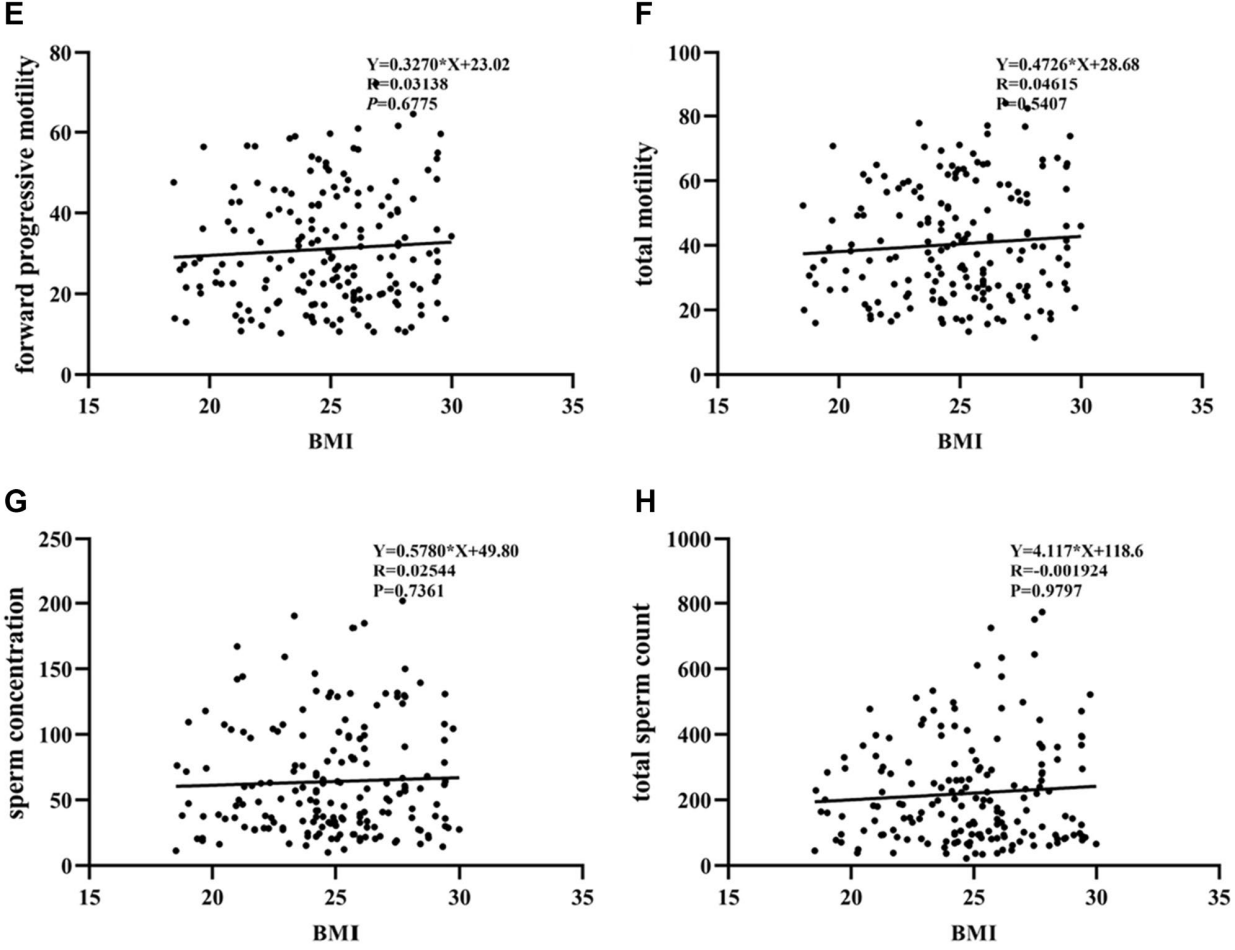
Correlation between BMI and sperm quality in men with enterotypes P and B. (**A**) Correlation between BMI and forward progressive sperm motility in men with enterotype P. (**B**) Correlation between BMI and total sperm motility in men with enterotype P. (**C**) Correlation between BMI and sperm concentration in men with enterotype P. (**D**) Correlation between BMI and total sperm count in men with enterotype P. (**E**) Correlation between BMI and forward progressive sperm motility in men with enterotype B. (**F**) Correlation between BMI and total motility in men with enterotype B. (**G**) Correlation between BMI and sperm concentration in men with enterotype B. (**H**) Correlation between BMI and total sperm count in men with enterotype B. Abbreviations: BMI, body mass index.

First, a rate of forward progressive sperm motility (Fig. 2A), total sperm motility (Fig. 2B), a sperm concentration (Fig. 2C) and a total sperm count (Fig. 2D) were decreasing with an increasing values of BMI in patients with enterotype P, but not in patients with enterotype B (Fig. 2E,F,G,H). Specifically, as shown in Table 4, in enterotype P group, overweight/obese was a risk factor for a decreased rate of forward progressive sperm motility (OR 3.350; 95% CI 1.881–5.966; P < 0.001) and a decreased rate of total sperm motility (OR 4.298; 95% CI 2.365–7.809; P < 0.001). On the contrary, in enterotype B group, no significant difference between overweight/obese and sperm motility was found (Supplementary Table 3). Second, in enterotype P, overweight/obese was still an independent risk factor for decreased rate of forward progressive sperm motility (OR 3.131; 95% CI 1.749–5.607; P < 0.001) after adjusting for FSH, and for decreased rate of total sperm motility (OR 3.387; 95% CI 1.804–6.357; P < 0.001) after adjusting for FSH and TT (Table 4). It remains an independent factor after adjusting for FSH, LH, TT and age (Supplementary Table 4).

**Table 4:** Logistic regression analysis of risk factors for asthenospermia in men with enterotype P.

	**Rate of forward progressive motility**		***P***	**Rate of total motility**		*P*	**Total sperm count**		***P***	**Sperm concentration**		***P***
	< 32% motile	Normal		< 40% motile	Normal		< 39 million	Normal		< 15 million/mL	Normal	
	N = 132	N = 97		N = 122	N = 107		N = 4	N = 225		N = 7	N = 222	
**Age**												
< 40	107	85	0.182	99	93	0.237	3	186	0.539	5	187	0.316
≥ 40	25	12		23	14		1	39		2	35	
**BMI, *N***												
< 24	28	46	** < 0.001**	22	52	** < 0.001**	1	73	1.000	3	71	0.684
≥ 24	104	51		100	55		3	152		4	151	
**Sex hormone**												
FSH	4.59(3.46–6.61)	4.09(3.14–5.64)	**0.036**	4.59(3.46–6.61)	4.09(3.14–5.64)	**0.005**	8.69(6.03–13.27)	4.37(3.27–6.04)	**0.009**	7.56(3.15–14.42)	4.40(3.31–6.04)	0.092
LH	3.26(2.29–4.28)	3.09(2.45–4.27)	0.737	3.26(2.29–4.28)	3.09(2.45–4.27)	0.508	5.70(3.21–7.74)	3.17(2.30–4.26)	0.050	4.92(3.31–7.40)	3.08(2.29–4.17)	**0.009**
E_2_	33.69(25.49–42.47)	35.30(28.12–42.38)	0.285	33.69(25.49–42.47)	35.30(28.12–42.38)	0.841	30.45(22.21–45.92)	34.31(26.85–42.42)	0.578	32.76(28.13–34.86)	34.46(26.79–42.44)	0.586
PRL	6.28(4.86–7.89)	6.52(4.67–8.25)	0.716	6.28(4.86–7.89)	6.52(4.67–8.25)	0.506	6.61(6.41–7.17)	6.29(4.71–8.18)	0.623	6.48(4.86–7.31)	6.30(4.70–8.21)	0.956
TT	3.51(2.61–4.41)	3.81(3.00–4.91)	0.086	3.51(2.61–4.41)	3.81(3.00–4.91)	**0.001**	4.59(2.91–5.88)	3.58(2.85–4.50)	0.293	5.00(2.49–6.17)	3.58(2.86–4.44)	0.177

Curde OR	**Rate of forward progressive motility**	***P***	**Rate of total motility**	***P***	**Total sperm count**	***P***	**Sperm concentration**	***P***				
**BMI**	3.350(1.881–5.966)	** < 0.001**	4.298(2.365–7.809)	** < 0.001**								
**Sex hormone**												
FSH	0.869(0.772–0.977)	**0.019**	0.819(0.722–0.929)	**0.002**	0.848(0.732–0.982)	**0.027**						
LH							0.581(0.403–0.840)	**0.004**				
E_2_												
PRL												
TT			1.300(1.072–1.577)	**0.008**								

Adjusted OR	**Rate of forward progressive motility**	***P***	**Rate of total motility**	***P***	**Total sperm count**	***P***	**Sperm concentration**	***P***				
**BMI**	3.131(1.749–5.607)	** < 0.001**	3.387(1.804–6.357)	** < 0.001**								
**Sex hormone**												
FSH	0.886(0.785–1.002)	0.053	0.817(0.714–0.936)	**0.003**								
LH												
E_2_												
PRL												
TT			1.212(0.988–1.487)	0.066								

BMI, body mass index; FSH, follicle-stimulating hormone; LH, luteinizing hormone; E_2_, estradiol; TT, total testosterone; PRL, prolactin. Median (interquartile range) are shown. The Mann–Whitney U test of nonparametric coefficients was used for non-normally distributed data. Significant values are bold.

### Prediction Performance of enterotypes for categorizing asthenospermia

**Figure 3:**
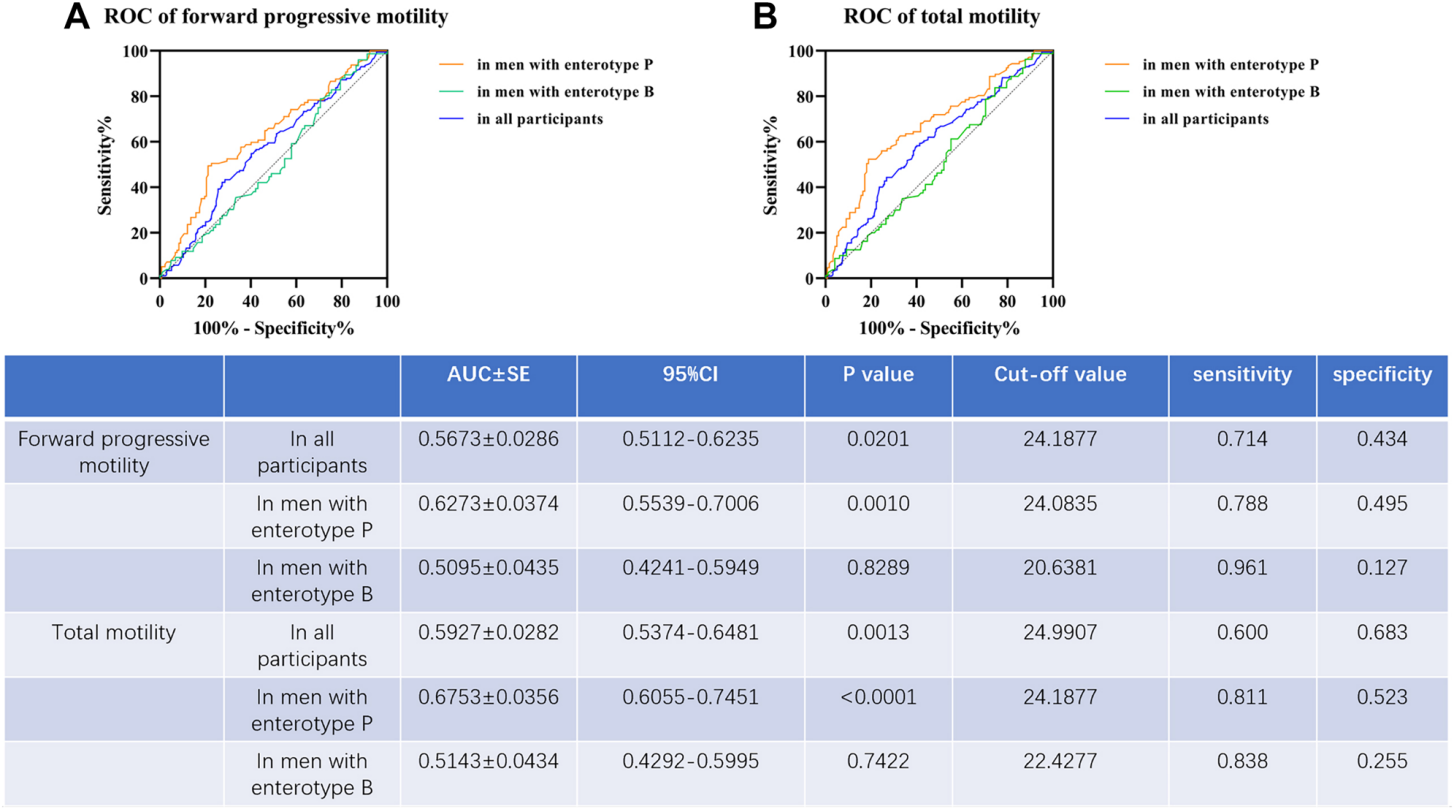
Diagnostic performance of BMI with respect to asthenospermia incidence. (**A**) Diagnostic potential of enterotypes in predicting the incidence of decreased forward progressive sperm motility. (**B**) Diagnostic potential of enterotypes in predicting the incidence of decreased total sperm motility. Abbreviations: AUC, area under curve; BMI, body mass index.

As mentioned above, in the enterotype P group, an increasing value of BMI was strongly associated with a decreasing sperm motility. Thus, with the help of ROC curves, the correlation between BMI and asthenospermia in patients with enterotype P were confirmed. In enterotype P group, BMI resulted in an area under the curve (AUC) of 62.7% and a moderate sensitivity (78.8%) and specificity (49.5%) for a decreased rate of forward progressive sperm motility (Fig. 3A). Similarly, the BMI levels also resulted in the higher AUC of 67.5%, with a sensitivity (81.1%) and a highest specificity (52.3%) for a decreased rate of total motility in men with enterotype P (Fig. 3B). In contrast, BMI showed poor prediction performance for asthenospermia in men with enterotype B (Fig. 3A,B).

## Discussion

To the best of our knowledge, this study is tried to use enterotypes as a population-based individualized classification index to investigate the correlation between BMI and asthenospermia. A correlation between obesity and asthenospermia in all participants was observed. Similar results were obtained in previous study. In a series of studies in Asian population, the results tend to be consistent. Obesity has a negative impact on sperm quality^5,15,16^. In a study on Chinese sperm donors, obesity was found to be significantly associated with a 4.2%, 3.9%, and 3.6% reduction in sperm volume, total sperm count, and total motile sperm count, respectively^5^. A cross-sectional study in Iran showed that total sperm count and sperm motility in overweight and obese men were significantly lower than those in men with normal BMI^16^. In a study based on data from infertility clinics in India, Ramaraju et al.^15^ found that obese men were more likely to have asthenospermia and oligospermia. Nerverthelss, different results were also yielded. Several studies in Europe have shown that men with excess body weight do not have considerable sperm motility problems^8,17–19^. A prospective cohort study in Netherlands showed that sperm quality was not statistically significantly affected by BMI in male partners in subfertile couples^8^. Similar results were also described in Danish and Austrian populations^17–19^. This may be related to factors such as population and region, and it is still necessary to expand the sample and conduct in-depth research.

Our results also showed that obesity was independent risk factor for poor sperm motility only in enterotype P but not enterotype B, and BMI could predict the risk of asthenospermia in the enterotype P group but not in the enterotype B group. Arumugam et al. first introduced the concept of “enterotype”, that the gut microbiome of different individuals can be divided into two enterotypes according to the dominant bacteria genera^14^, and it has been found to be associated with long-term diets, remaining stable over a long duration^20,21^. An enriched Prevotella enterotype, characterized by a higher content of short-chain fatty acids and genes for polysaccharide degradation, was demonstrated among Egyptian teenagers^22^, while Bacteroides enterotype was more common in in American teenagers, whose intestines were rich in amino acids and lipid metabolism^20^. Arumugam suggested that this well-balanced host-microbial symbiotic states might respond differently to diet and drug intake. This hypothesis has been supported by a study in which people with the *Prevotella* and *Bacteroides* enterotypes respond differently to dietary fiber^23^. These results uncover a potentially intimate association between enterotypes and metabolism. Based on the above information, it can be speculated that there are potential differences in metabolism state and susceptibility of disorders between enterotypes. Since the reason why sperm quality associates with BMI in only a subset of men remains controversial, our study raises an intriguing mechanistic hypothesis that this may due to underlying changes in the microbiome. Our study showed that obesity was a risk factor for decreased rate of forward progressive sperm motility and decreased rate of total sperm motility in enterotype P other than enterotype B. Enterotype P was predicted with lower activity of bile acid biosynthesis^24^*.* Farnesoid X Receptor (FXR)(one of the receptor of bile acid)has been demonstrated to be expressed in the male genital tract, and FXR agonist could improve the metabolic state^25^. An improved metabolic status correlates positively to sperm quality^7^, and associates with improved mitochondrial ultrastructure and dynamics and reduced superoxide production^26^, whichare essential to the spermatogenesis process and sperm quality. Therefore, decreased sperm quality in male with enterotype P may be associated with metabolic disorders.

What’s more, sperm quality has been demonstrated to be related to male genital tract inflammation^27^. Previous studies have reported that genital tract inflammation are associated with asthenozoospermia^27,28^, and a study of leukocytospermic showed sperm motility is affected by inflammation^29^. The microbiome of male with leukocytospermic differed from that of normal population^29^. A Prevotella-dominated gut enterotype has been shown to increase inflammation level^30^. This suggests that gut microbes may increase the level of inflammation in the body, causing inflammation of the reproductive tract. Thus we speculate that sperm of enterotype P male may also be affected by increasing inflammation in the intestine and reproductive tract. Besides, there were also strong evidence suggest a role for sex steroids during metabolic syndrome and visceral obesity, for instance, estrogen acts as pro-inflammatory factor while androgen as anti-inflammatory factor^31^. In our subjects, we compared the sex hormone levels of two obese men with enteric type and found no difference (Supplementary Table 5). This may be because our study included asthenozoospermia and normal controls rather than patients or models with overt metabolic disease.

The results from this study are promising and warrant more research, and it is worth noting that our study has limitations. Despite controlling for age and other sex hormone covariates, it remains to be further clarified whether the phenomena associated with intestinal microbiota demonstrated here are truly causal to male reproductive disease. In addition, though BMI is a widely used indicator for assessing obesity and leanness, it remains unclear whether using BMI alone is a proxy for overall health. We had no direct measure of visceral fat, which is a better measure of obesity.

In conclusion, obesity is independent risk factor of asthenozoospermia, especially in male with enterotype P. The difference that we observed across different enterotypes added a new potential factor between the relationship between sperm quality and obesity, which needs to be further elucidated. The use of enterotype to classify men with decreased sperm quality may help investigate individualized potential diagnostic and therapeutic opportunities in male health.

## Materials and methods

### Ethical statement

This study was conducted in accordance with the Code of Ethics and the 1975 Declaration of Helsinki. The study protocol was approved by the Ethics Committee of the Shengjing Hospital of China Medical University (Reference No. 2017PS190K). Informed consent was obtained from all participants.

### Participants

All the participants recruited at Shengjing Hospital of China Medical University and Jinghua Hospital of Shenyang from October 2020 to April 2021. Inclusion criteria is age 18–49 years; patients who had taken antibiotics and probiotics within 1 month prior to the study were excluded. Blood sample was collected and measured on the day of clinic. Sperm sample was obtained after abstain from ejaculation for 3–7 days. Fecal sample was taken and stored on the same day or the next day of sperm collection. Sperm motility was divided into three categories: progressive, including rapidly and slowly progressive, non-progressive, and immotile according to the World Health Organization (WHO) criteria. The diagnostic criteria for asthenospermia are based on WHO laboratory manual for the examination and processing of human sperm, and men who meet the criteria are diagnosed with asthenospermia, implying that the percentage of sperm forward movement in sperm is less than 32%, and two or more sperm analyses are recommended. Patients with obstructive and non-obstructive azoospermia were excluded. Ultimately, a total of 407 men were included in the study.

### Parameter measurements

BMI was calculated by dividing body weight (kg) by height (m) squared. Levels of sex hormones—follicle-stimulating hormone (FSH), luteinizing hormone, estradiol, total testosterone (TT), and progestin—were measured with a chemiluminescence immunoassay. Sperm samples were collected in a sterilized container through masturbation in a dedicated sperm collection room; condoms or lubricants were not used. Sperm analysis was conducted soon after liquefaction (< 60 min). Sperm parameters including sperm concentration, total sperm count, and the percentage of each motility category of sperm were measured with WLJY9000, an instrument of computer-aided sperm analysis. Normal sperm reference values were determined according to WHO criteria. Throughout the study, external quality control was performed.

### Extraction of microbiota DNA and quantitative polymerase chain reaction (qPCR) amplification

The fecal samples were stored at −20 °C after collected and transported to the research center on dry ice within 24 h of collection, where they were stored at −80 °C until DNA was extracted. The TIANamp stool DNA kit (Tiangen Biotech [Beijing] Co., Ltd, Beijing, China) was used to extract the bacterial DNA from stool samples according to the manufacturer’s protocol. DNA concentration was measured with a Qubit 2.0 fluorometer (Life Technologies, Carlsbad, CA, USA). The concentration and purity of the recovered DNA fragments were determined via agarose gel electrophoresis and ultraviolet spectrophotometry. The OD260/OD280 ratio was set between 1.7 and 1.9. qPCR was performed with the Detect Genus Gut Microbes Detection Kit and PCR-Fluorescence Probe (TiangenBiotech [Beijing] Co., Ltd). The *Prevotella* and *Bacteroides* primers were designed according to the results of the qPCR test (Supplementary Table 1). The amplification procedure was as follows: Samples were heated at 95 °C for five minutes, then shifted to 45 15 s cycles at 95 °C and one 40 s cycle at 56 °C. qPCR was performed on the ABI 7500 Real-Time PCR system (Applied Biosystems, Waltham, MA, USA), and the amplification conditions were set to achieve the best response.

### Statistical methods

Enterotypes were identified by plotting the log-transformed abundance of *Bacteroides* versus the log-transformed abundance of *Prevotella*, which were calculated using the *diptest* package in R (The R Project for Statistical Computing, Vienna, Austria). A histogram plotting frequency of the log-transformed abundance of *Prevotella*/*Bacteroides* (P/B) was obtained using GraphPad Prism 6 (GraphPad Software, San Diego, CA, USA). The Mann–Whitney *U* test of nonparametric coefficients was used to identify any significant differences in the clinical indices between the groups. The data in the tables were presented as median (interquartile range) and as histograms. Univariate and multivariate logistic regression analyses were carried out using SPSS statistics version 23.0 (IBM, Armonk, NY, USA). The receiver operating characteristic (ROC) curve was modeled using the GraphPad Prism 6 (GraphPad Software).

## Data availability

The data that support the findings of this study are available in fig share at https://doi.org/10.6084/m9.figshare.19386542.v1, reference number 25.

## Supplementary Information

Supplementary Information.
