# Supplementary material for: Enterotypes in asthenospermia patients with obesity
Source: Sci Rep. 2022 Oct 10;12:16993. doi: 10.1038/s41598-022-20574-0 (PMC9550853; doi:10.1038/s41598-022-20574-0)
Supplement: Supplementary file 1 — Supplementary Information. [file 41598_2022_20574_MOESM1_ESM.docx]

**Supplemental table 1** The primer and TaqMan probe sequences

|  | | Sequence | Reference |
| --- | --- | --- | --- |
| *Prevotella* | Up stream | CCAGCCAAGTAGCGTGCA | (Martin*, et al.*,2002) |
|  | Down stream | TGGACCTTCCGTATTACCGC |  |
|  | Probe | AATAAGGACCGGCTAATTCCGTGCCAG |  |
| *Bacteroides* | Up stream | VGATGGGGATGCGTTCCATTAG | (Kurina*, et al.*,2020) |
|  | Down stream | CATCCTTCACGCTACTTGGCTGG |  |
|  | Probe | CGATGGATAGGGGTTCTGAGAGGAAGGTC |  |

Kurina I., A. Popenko, N. Klimenko, S. Koshechkin, L. Chuprikova, M. Filipenko, A. Tyakht and D. Alexeev.Development of qPCR platform with probes for quantifying prevalent and biomedically relevant human gut microbial taxa.*Mol Cell Probes*.Aug,2020;101570

Martin F. E., M. A. Nadkarni, N. A. Jacques and N. Hunter.Quantitative microbiological study of human carious dentine by culture and real-time PCR: association of anaerobes with histopathological changes in chronic pulpitis.*J Clin Microbiol*.May,2002;**5**:1698-704

**Supplemental table 2 Logistic regression analysis of risk factors for asthenospermia adjusting by age, FSH, LH and TT.**

| curde OR | **rate of forward progressive motility** | ***P*** | **rate of total motility** | ***P*** | **total sperm count** | ***P*** | **sperm concentration** | ***P*** |
| --- | --- | --- | --- | --- | --- | --- | --- | --- |
| **age** | 1.482(0.829-2.652) | 0.184 | 1.349(0.766-2.377) | 0.300 | 1.349(0.284-6.408) | 0.706 | 1.349(0.284-6.408) | 0.706 |
| **BMI** | 1.849(1.218-2.807) | **0.004** | 2.046(1.346-3.111) | **0.001** | 0.865(0.249-3.008) | 0.820 | 0.865(0.249-3.008) | 0.820 |
| **sex hormone** |  |  |  |  |  |  |  |  |
| FSH | 0.898(0.825-0.979) | **0.014** | 0.885(0.812-0.964) | **0.005** | 0.825(0.726-0.938) | **0.003** | 0.785(0.681-0.904) | **0.001** |
| LH | 0.972(0.862-1.096) | 0.641 | 1.000(0.889-1.125) | 1.000 | 0.801(0.639-1.003) | 0.054 | 0.759(0.609-0.946) | **0.014** |
| TT | 1.074(0.938-1.230) | 0.301 | 1.111(0.969-1.273) | 0.133 | 0.798(0.585-1.088) | 0.153 | 0.788(0.581-1.070) | 0.127 |
| adjusted OR | **rate of forward progressive motility** | ***P*** | **rate of total motility** | ***P*** | **total sperm count** | ***P*** | **sperm concentration** | ***P*** |
| **age** | 1.352(0.742-2.464) | 0.324 | 1.192(0.661-2.149) | 0.559 | 0.843(0.133-5.341) | 0.856 | 0.733(0.102-5.285) | 0.758 |
| **BMI** | 1.776(1.136-2.774) | **0.012** | 1.913(1.224-2.991) | **0.004** | 0.775(0.201-2.980) | 0.711 | 0.681(0.172-2.707) | 0.586 |
| **sex hormone** |  |  |  |  |  |  |  |  |
| FSH | 0.885(0.799-0.981) | **0.020** | 0.852(0.767-0.946) | **0.003** | 0.812(0.689-0.957) | **0.013** | 0.777(0.650-0.929) | **0.006** |
| LH | 1.073(0.922-1.249) | 0.364 | 1.134(0.974-1.320) | 0.106 | 1.016(0.719-1.435) | 0.929 | 0.992(0.721-1.365) | 0.961 |
| TT | 0.986(0.914-1.063) | 0.909 | 1.016(0.871-1.185) | 0.844 | 0.821(0.598-1.126) | 0.221 | 0.824(0.603-1.126) | 0.223 |

**Supplemental table 3 Logistic regression analysis of risk factors for asthenospermia in men with enterotype B**

|  | **rate of forward progressive motility** | | ***P*** | **rate of total motility** | |  | **total sperm count** | | ***P*** | **sperm concentration** | | ***P*** |
| --- | --- | --- | --- | --- | --- | --- | --- | --- | --- | --- | --- | --- |
| **BMI, *N*** | <32% motile | normal |  | <40% motile | normal |  | <39 million | normal |  | <15 million/mL | normal |  |
| <24 | 36 | 25 | 0.739 | 35 | 26 | 0.653 | 3 | 58 | 0.692 | 1 | 60 | 1.000 |
| ≥24 | 66 | 51 |  | 63 | 54 |  | 4 | 113 |  | 3 | 114 |  |
| **sex hormone** |  |  |  |  |  |  |  |  |  |  |  |  |
| FSH | 4.73(3.41-6.06) | 4.58(3.62-5.56) | 0.554 | 4.70(3.37-5.95) | 4.67(3.62-5.97) | 0.965 | 7.18(5.12-8.96) | 4.56(3.41-5.87) | **0.004** | 6.98(6.55-8.06) | 4.62(3.41-5.88) | **0.009** |
| LH | 3.27(2.21-4.22) | 2.98(2.13-4.25) | 0.365 | 3.12(2.18-4.12) | 3.06(2.22-4.38) | 0.946 | 4.18(2.54-4.91) | 3.08(2.18-4.14) | 0.281 | 4.15(3.41-4.34) | 3.07(2.18-4.22) | 0.176 |
| E_2_ | 31.63(23.69-39.27) | 34.74(20.20-41.50) | 0.367 | 31.98(23.56-39.71) | 33.53(21.02-39.99) | 0.765 | 24.16(23.13-47.64) | 33.53(22.29-39.81) | 0.608 | 23.60(19.54-26.99) | 33.53(23.33-39.88) | 0.107 |
| PRL | 6.46(4.91-8.37) | 6.19(4.78-8.23) | 0.520 | 6.61(5.02-8.31) | 6.19(4.63-8.32) | 0.397 | 5.88(4.38-8.19) | 6.45(4.91-8.31) | 0.452 | 6.00(4.60-9.15) | 6.45(4.90-8.31) | 0.895 |
| TT | 3.65(2.73-4.39) | 3.37(2.70-4.47) | 0.489 | 3.77(2.73-4.47) | 3.31(2.70-4.14) | 0.184 | 3.57(3.26-5.62) | 3.56(2.69-4.38) | 0.205 | 4.15(2.88-5.50) | 3.53(2.71-4.39) | 0.450 |

Abbreviations: BMI, body mass index; FSH, follicle-stimulating hormone; LH, luteinizing hormone; E_2_, estradiol; TT, total testosterone; PRL, prolactin; P/B, *Prevotella*/*Bacteroides*. Median (interquartile range) are shown. The Mann–Whitney U test of nonparametric coefficients was used for non-normally distributed data.

**Supplemental table 4 Logistic regression analysis of risk factors for asthenospermia adjusting by age, FSH, LH and TT in men with enterotype P.**

| curde OR | **rate of forward progressive motility** | ***P*** | **rate of total motility** | ***P*** | **total sperm count** | ***P*** | **sperm concentration** | ***P*** |
| --- | --- | --- | --- | --- | --- | --- | --- | --- |
| **age** | 1.655(0.786-3.486) | 0.185 | 1.543(0.750-3.177) | 0.239 | 1.750(0.177-17.300) | 0.632 | 2.137(0.399-11.456) | 0.375 |
| **BMI** | 3.350(1.881-5.966) | **<0.001** | 4.298(2.365-7.809) | **<0.001** | 1.441(0.147-14.091) | 0.754 | 0.627(0.137-2.876) | 0.548 |
| **sex hormone** |  |  |  |  |  |  |  |  |
| FSH | 0.869(0.772-0.977) | **0.019** | 0.819(0.722-0.929) | **0.002** | 0.848(0.732-0.982) | **0.027** | 0.787(0.666-0.930) | **0.005** |
| LH | 1.031(0.868-1.226) | 0.726 | 1.034(0.871-1.228) | 0.699 | 0.571(0.369-0.884) | **0.012** | 0.581(0.403-0.840) | **0.004** |
| TT | 1.173(0.981-1.401) | 0.080 | 1.300(1.072-1.577) | **0.008** | 0.825(0.522-1.304) | 0.410 | 0.799(0.567-1.126) | 0.200 |
| adjusted OR | **rate of forward progressive motility** | ***P*** | **rate of total motility** | ***P*** | **total sperm count** | ***P*** | **sperm concentration** | ***P*** |
| **age** | 1.395(0.629-3.092) | 0.413 | 1.269(.566-2.845) | 0.564 | 1.319(0.080-21.730) | 0.847 | 1.731(.193-15.527) | 0.624 |
| **BMI** | 2.917(1.572-5.414) | **0.001** | 3.460(1.825-6.559) | **<0.001** | 0.783(0.062-9.976) | 0.851 | 0.229(0.031-1.692) | 0.149 |
| **sex hormone** |  |  |  |  |  |  |  |  |
| FSH | 0.841(0.729-0.970) | **0.018** | 0.765(0.653-0.895) | **0.001** | 0.913(0.756-1.103) | 0.346 | 0.821(0.673-1.000) | **0.050** |
| LH | 1.184(0.944-1.484) | 0.144 | 1.241(0.980-1.573) | 0.074 | 0.605(0.338-1.083) | 0.091 | 0.658(0.382-1.133) | 0.131 |
| TT | 1.033(0.840-1.270) | 0.758 | 1.146(0.924-1.421) | 0.215 | 1.015(0.635-1.621) | 0.952 | 0.953(0.657-1.383) | 0.801 |

**Supplemental table 5 Comparison of sex hormone between enterotype B and enterotype P in men with BMI ≥24 kg/m^2^.**

|  | **Enterotype B**  **N=117** | **Enterotype P**  **N=155** | ***P*** |
| --- | --- | --- | --- |
| FSH | 4.60(3.37-5.91) | 4.50(3.46-6.45) | 0.548 |
| LH | 2.89(2.22-4.13) | 3.25(2.21-4.27) | 0.829 |
| E_2_ | 32.64(23.88-39.61) | 34.05(27.26-41.48) | 0.086 |
| PRL | 6.45(5.04-8.50) | 6.29(4.57-8.14) | 0.323 |
| TT | 3.13(2.62-4.08) | 3.37(2.53-4.20) | 0.577 |
